# Early neurological improvement as a dynamic predictor for 90-day functional outcome in acute ischemic stroke: a prospective cohort study

**DOI:** 10.3389/fmed.2026.1757614

**Published:** 2026-03-09

**Authors:** Zhiyuan Chu, Xinzheng Fu, Zhouming Ren, Tian Le, Xuyan Zhang, Yueyue Zhang, Wei Yao

**Affiliations:** Haining People’s Hospital, Haining, China

**Keywords:** acute ischemic stroke, early neurological improvement, functional outcome, NIHSS, nomogram, prognosis

## Abstract

**Background:**

Early neurological improvement (ENI) within the first 24 h after acute ischemic stroke (AIS) has been proposed as a rapid dynamic predictor for treatment response. However, the prognostic value of ENI for 90-day functional recovery in real-world clinical practice remains uncertain, especially in heterogeneous stroke populations receiving mixed reperfusion treatments. We aimed to evaluate the association between 24-h neurological change and 90-day functional outcome in a contemporary single-center AIS cohort.

**Methods:**

We conducted a prospective observational cohort study including 200 consecutive AIS patients between January 2023 and December 2024. Baseline demographic, vascular risk factor, clinical, laboratory, imaging, and treatment variables were collected at admission. ENI was defined as the change in NIHSS between baseline and 24 h (ΔNIHSS = NIHSS_baseline–NIHSS_24h). Two logistic regression models were developed: Model 1, using only baseline clinical and imaging variables, and Model 2, which incorporated ΔNIHSS as a dynamic predictor. Discrimination was evaluated using the area under the receiver operating characteristic curve (AUC), and calibration was assessed using bootstrap-corrected calibration plots. Nomograms were constructed for bedside application.

**Results:**

Among 200 patients, 118 (59.0%) achieved good functional outcome at 90 days (mRS 0–2). In Model 1, age, baseline NIHSS, and ASPECTS were independently associated with 90-day outcomes, whereas hypertension was negatively associated. Model 1 demonstrated strong discrimination (AUC 0.863). While discrimination reached a prognostic plateau (AUC 0.863 vs. 0.855), the incorporation of ΔNIHSS significantly optimized model calibration, reducing the mean absolute prediction error by 47% (0.051–0.027). This indicates that the dynamic model provides substantially more accurate probability estimates for individual patients. Greater early neurological improvement was independently associated with good outcome (adjusted OR per 5-point ΔNIHSS increase, 1.48; 95% CI 1.11–1.97). Corresponding ROC curves, calibration plots, and nomograms for both models are presented.

**Conclusion:**

Early neurological improvement within 24 h after AIS serves as a reliable and rapid dynamic predictor for 90-day functional recovery. While baseline clinical and imaging variables provide strong prognostic value, incorporating early neurological change enhances model calibration and clinical usefulness. This dynamic paradigm supports integrating short-term neurological change into prognostic assessment and individualized post-stroke care.

## Introduction

Acute ischemic stroke (AIS) remains a leading cause of long-term disability and mortality worldwide, despite substantial advances in reperfusion therapy over the past decade (1). Early identification of patients likely to achieve favorable outcomes is essential for guiding treatment decisions, allocating post-acute rehabilitation resources, and counseling families. Traditional prognostic factors—such as age, baseline neurological severity, and early ischemic changes on neuroimaging—provide valuable but incomplete insight into individual trajectories of recovery (2, 3). These variables capture the initial physiological insult but fail to reflect treatment responsiveness or early dynamic changes in cerebral function.

Recent evidence has emphasized the prognostic significance of early neurological improvement (ENI) as a rapid, clinically observable indicator of treatment response following intravenous thrombolysis or mechanical thrombectomy (4–7). ENI, commonly defined as improvement in the NIHSS score within the first 24 h, is strongly associated with reperfusion, reduced infarct growth, and improved functional outcomes (8). However, most existing studies have focused exclusively on highly selected cohorts undergoing endovascular therapy, limiting generalizability to the broader AIS population where mixed treatment strategies are used and early improvement may arise from multiple mechanisms, including partial reperfusion, collateral recruitment, and neuroplastic compensation (9).

Moreover, the prognostic utility of ENI in real-world, non-trial settings remains incompletely understood. Heterogeneous patient characteristics, variable time-to-treatment intervals, and differences in acute stroke workflows introduce complexities that may alter the predictive value of early neurological change. A recent meta-analysis suggested that ENI is a strong surrogate endpoint for 90-day modified Rankin Scale (mRS) outcomes, but highlighted substantial inconsistency in definitions, analytic methods, and population selection (10). Contemporary guidelines have not formally incorporated ENI into prognostic recommendations, partly due to the lack of prospective evidence evaluating its incremental value over established baseline markers (11).

To address this knowledge gap, dynamic prediction models incorporating both baseline data and early post-treatment change have been proposed as a means to refine prognostication in AIS (12). However, few studies have directly compared baseline-only versus dynamic neurological models using standardized statistical evaluation frameworks, including discrimination, calibration, and clinical interpretability through nomograms. Furthermore, the extent to which ENI improves predictive performance in an unselected clinical cohort—beyond what is already captured by age, initial NIHSS, ASPECTS, and treatment variables—remains uncertain.

Therefore, in this prospective single-center cohort study, we aimed to comprehensively evaluate the prognostic utility of early neurological change following acute ischemic stroke. Specifically, our objectives were to: (1) quantify the association between 24-h neurological improvement and 90-day functional recovery (13); (2) develop and compare two multivariable logistic regression models—one incorporating only baseline bedside clinical variables and a second dynamic model that additionally included early neurological improvement; and (3) assess the discrimination, calibration, and bedside applicability of both models using receiver operating characteristic analysis, bootstrap-corrected calibration methods, and nomogram-based individualized prediction (14). We hypothesized that integrating early neurological change into prognostic modeling would enhance predictive accuracy and provide a clinically practical dynamic predictor for long-term functional outcomes.

## Materials and methods

### Study design and setting

We conducted a prospective, observational cohort study at a comprehensive stroke center in Haining People’s Hospital from January 2023 through December 2024. The study protocol adhered to the principles of the Declaration of Helsinki and was approved by the institutional ethics committee (Approval No. 2024-016). The requirement for written informed consent was waived by the institutional ethics committee due to the observational nature of the study. Clinical data collection followed standardized institutional stroke pathways and national registry guidelines.

### Participants

All consecutive adults aged ≥ 18 years who presented with acute ischemic stroke (AIS) confirmed by CT or MRI were screened for eligibility. Exclusion criteria were: (1) hemorrhagic stroke; (2) stroke mimics (e.g., seizure, functional disorder); (3) pre-stroke mRS > 2; (4) incomplete NIHSS assessment at baseline or at 24 h; or (5) missing 90-day outcome assessment. A total of 200 patients were included in the final analysis. Patients were excluded if NIHSS assessment at baseline or 24 h was incomplete, or if 90-day outcome data were unavailable.

### Baseline assessments

Demographic variables (age, sex, BMI), vascular risk factors (hypertension, diabetes, atrial fibrillation, hyperlipidemia, smoking, prior stroke, coronary artery disease), and admission vital signs (blood pressure, glucose, creatinine) were recorded at presentation. Neurological severity was assessed using the National Institutes of Health Stroke Scale (NIHSS) performed by certified neurologists. Early ischemic changes were quantified using the Alberta Stroke Program Early CT Score (ASPECTS) on baseline NCCT or DWI, interpreted by two independent neuroradiologists blinded to outcomes, with discrepancies resolved by consensus.

### Early neurological improvement

Early neurological improvement (ENI) was quantified as the absolute change in NIHSS score from baseline to 24 h:


$$\Delta \,\mathrm{NIHSS}={\mathrm{NIHSS}}_{\mathrm{baseline}}-{\mathrm{NIHSS}}_{24\,h}$$

Positive ΔNIHSS represented improvement; negative values represented deterioration. Early neurological deterioration (END) was defined as a ≥ 4-point increase in NIHSS within 24 h, consistent with prior studies.

### Treatment variables

Patients received intravenous thrombolysis (rtPA) based on guideline-recommended criteria and time window. Mechanical thrombectomy (MT) was performed for eligible large vessel occlusion (LVO) patients according to contemporary AHA/ASA recommendations (11). Procedural variables, including door-to-needle time, door-to-puncture time, occlusion location, and collateral grade, were collected. Hemorrhagic transformation and symptomatic intracranial hemorrhage (sICH) were adjudicated per ECASS-III criteria.

### Outcome measurement

The primary outcome was 90-day functional outcome, assessed using the modified Rankin Scale (mRS) via structured telephone or in-person interviews. Favorable outcome was defined as mRS 0–2.

### Model development

Two predefined multivariable logistic regression models were constructed:

#### Model 1 — baseline bedside model

Model 1, the baseline bedside prediction model, incorporated only variables available at the time of initial clinical evaluation. These included age, baseline neurological severity measured by the NIHSS, early ischemic changes quantified by ASPECTS, and major vascular risk factors such as hypertension, diabetes mellitus, and atrial fibrillation. In addition, the model accounted for acute reperfusion strategies by including indicators of intravenous thrombolysis (rtPA) and mechanical thrombectomy. All variables were selected a priori based on established clinical relevance and their consistent association with functional outcomes in previous stroke literature.

#### Model 2 — dynamic model incorporating ENI

Included all variables in Model 1 plus:

- ΔNIHSS (baseline–24h NIHSS)

This approach aligns with modern dynamic prediction frameworks advocated in stroke prognosis research (12).

### Statistical methods

Continuous variables were summarized as mean ± SD or median (IQR) and compared with *t*-tests or Wilcoxon rank-sum tests. Categorical variables were summarized as counts (%) and compared with χ^2^ or Fisher exact tests.

Logistic regression was used to estimate adjusted odds ratios (aORs) with 95% confidence intervals (CIs). Model discrimination was evaluated using area under the ROC curve (AUC). Calibration was assessed using bootstrap-corrected calibration curves generated via the *rms* package (300 resamples). Nomograms were developed for both models for individualized probability estimation.

ΔNIHSS was modeled as a continuous variable to preserve the full granularity of early neurological change and to avoid information loss associated with arbitrary categorization. This approach is consistent with prior prognostic modeling studies in acute ischemic stroke and reflects the clinically continuous nature of neurological recovery. For regression analyses, effect estimates were expressed per five-point increase in ΔNIHSS to enhance clinical interpretability and to avoid reporting minimal effect sizes per one-point change. All analyses were performed in R version 4.4.1.

## Results

### Patient characteristics

During the study period, 232 consecutive patients with acute ischemic stroke were screened for eligibility. Thirty-two patients were excluded, including 23 patients due to incomplete NIHSS assessment at baseline or 24 h and nine patients due to missing 90-day functional outcome data. After these exclusions, 200 patients were included in the final analysis. Patient inclusion and exclusion are summarized in Supplementary Figure 1.

Among 200 AIS patients, the mean age was 67.9 ± 10.8 years, and 45% were male. Hypertension (65%), diabetes (25%), atrial fibrillation (20%), and prior stroke (18%) were common. Median baseline NIHSS was 12 (IQR, 7–17), and median ASPECTS was 8 (IQR, 6–9). A total of 72 patients (36%) received rtPA and 48 (24%) underwent mechanical thrombectomy. Early neurological improvement varied widely (median ΔNIHSS, 3 points; IQR, 0–6). At 90 days, 118 patients (59%) achieved good functional outcome (Table 1).

**TABLE 1:** Baseline characteristics of the study population by 90-day functional outcome.

Characteristic	Total (*N* = 200)	Good outcome (*n* = 118)	Poor outcome (*n* = 82)	*P*-value
**Demographics**				
Age, y, mean ± SD	67.9 ± 10.8	65.1 ± 10.2	71.8 ± 9.8	<0.001
Male sex, no. (%)	90 (45.0)	58 (49.2)	32 (39.0)	0.14
BMI, kg/m^2^, mean ± SD	24.7 ± 3.2	24.9 ± 3.1	24.5 ± 3.4	0.46
**Vascular risk factors**				
Hypertension, no. (%)	130 (65.0)	68 (57.6)	62 (75.6)	0.008
Diabetes, no. (%)	50 (25.0)	23 (19.5)	27 (32.9)	0.03
Atrial fibrillation, no. (%)	40 (20.0)	18 (15.3)	22 (26.8)	0.05
Hyperlipidemia, no. (%)	70 (35.0)	43 (36.4)	27 (32.9)	0.6
Smoking history, no. (%)	62 (31.0)	38 (32.2)	24 (29.3)	0.67
Previous ischemic stroke, no. (%)	36 (18.0)	16 (13.6)	20 (24.4)	0.06
Coronary artery disease, no. (%)	28 (14.0)	13 (11.0)	15 (18.3)	0.16
**Clinical presentation**				
Baseline NIHSS, median (IQR)	12 (7–17)	9 (5–13)	17 (13–21)	<0.001
Systolic BP, mmHg, mean ± SD	154 ± 25	150 ± 23	160 ± 27	0.01
Diastolic BP, mmHg, mean ± SD	88 ± 14	86 ± 12	91 ± 15	0.03
Admission glucose, mmol/L, mean ± SD	7.8 ± 2.4	7.2 ± 2.1	8.7 ± 2.6	<0.001
Creatinine, μmol/L, median (IQR)	73 (62–88)	71 (60–84)	77 (68–95)	0.02
**Imaging variables**				
ASPECTS, median (IQR)	8 (6–9)	9 (7–10)	6 (4–8)	<0.001
LVO presence, no. (%)	72 (36.0)	32 (27.1)	40 (48.8)	0.002
**Treatment variables**				
IV rtPA, no. (%)	72 (36.0)	48 (40.7)	24 (29.3)	0.1
Mechanical thrombectomy, no. (%)	48 (24.0)	22 (18.6)	26 (31.7)	0.03
Door-to-needle time, min, median (IQR)	41 (32–55)	38 (30–50)	46 (36–64)	0.02
Door-to-puncture time, min, median (IQR)	84 (65–109)	79 (61–97)	93 (72–121)	0.03
**Early neurological change**				
ΔNIHSS (baseline–24 h), median (IQR)	3 (0–6)	6 (3–9)	0 (−2 to 2)	<0.001
Early neurological deterioration (END), no. (%)	18 (9.0)	2 (1.7)	16 (19.5)	<0.001
**Outcome**				
90-day mRS 0–2, no. (%)	118 (59.0)	–	–	–

### Univariable analyses

Patients with good outcomes were significantly younger (65.1 vs. 71.8 years), had lower baseline NIHSS, higher ASPECTS, lower prevalence of hypertension, and showed greater ΔNIHSS compared with those with poor outcomes (all *P* < 0.05). LVO patients were less likely to achieve good outcomes, although reperfusion treatments partially mitigated this association.

### Model 1: baseline bedside model

In the multivariable logistic regression model, several baseline clinical variables demonstrated significant independent associations with achieving a favorable 90-day functional outcome. Older age was strongly associated with decreased odds of good recovery (adjusted OR per 10-year increase, 0.61; 95% CI, 0.47–0.78; *P* < 0.001). Greater initial stroke severity also predicted poorer prognosis, with each five-point increase in baseline NIHSS corresponding to substantially lower odds of favorable outcome (aOR, 0.41; 95% CI, 0.31–0.55; *P* < 0.001). Conversely, higher ASPECTS values were associated with increased likelihood of good functional recovery (aOR per one-point increase, 1.33; 95% CI, 1.10–1.64; *P* = 0.005). In addition, hypertension was independently associated with lower odds of achieving functional independence at 90 days (aOR, 0.52; 95% CI, 0.29–0.91; *P* = 0.02). Treatment with rtPA or MT showed favorable trends but were not independent predictors in the multivariable model. This suggests that the prognostic benefit of reperfusion is largely mediated through the observed early neurological improvement (ΔNIHSS) and preserved brain tissue (ASPECTS), rather than the intervention itself.

**FIGURE 1:**
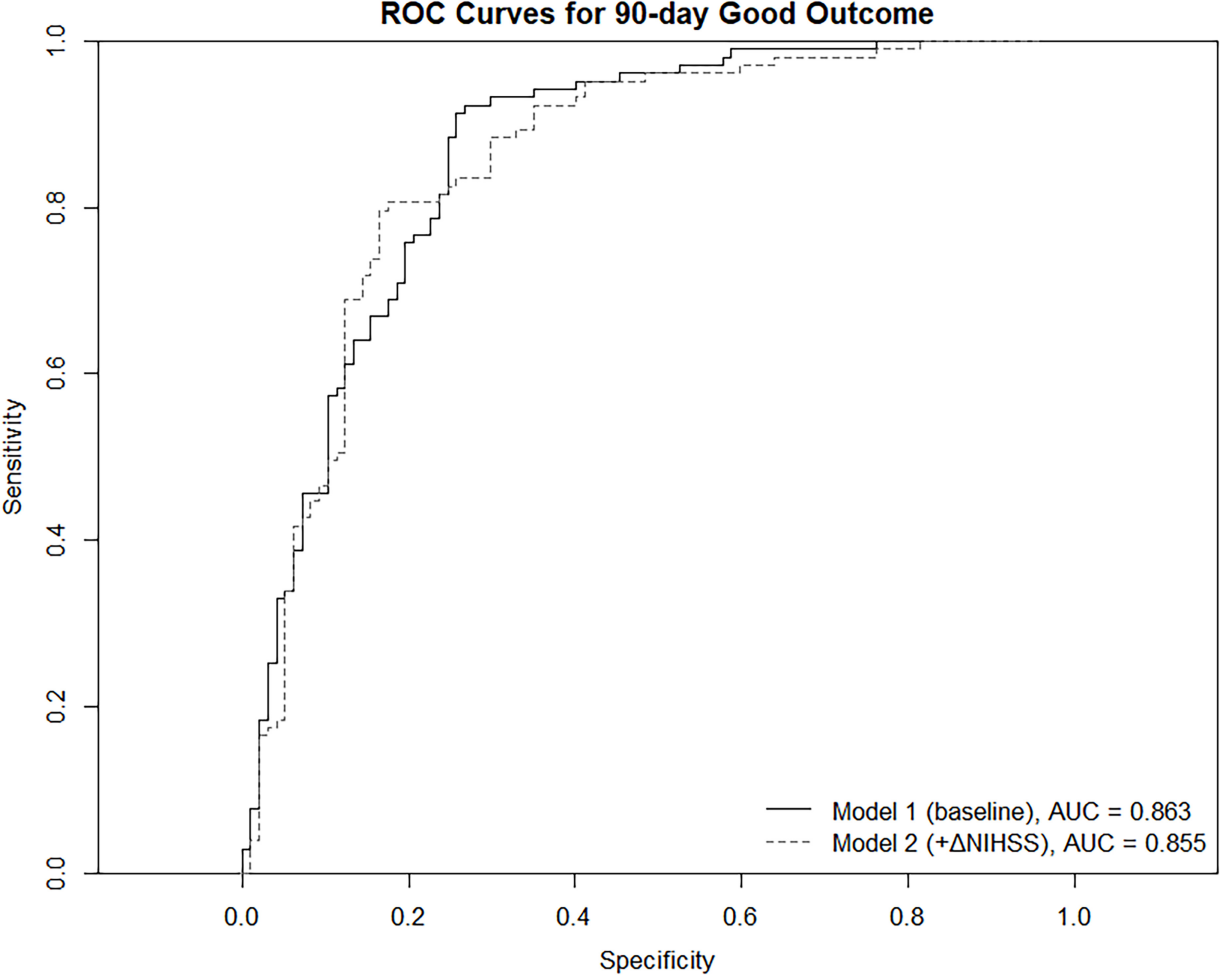
Receiver operating characteristic (ROC) curves comparing the discrimination performance of Model 1 (baseline model) and Model 2 (dynamic model incorporating ΔNIHSS) for predicting 90-day good functional outcome.

**FIGURE 2:**
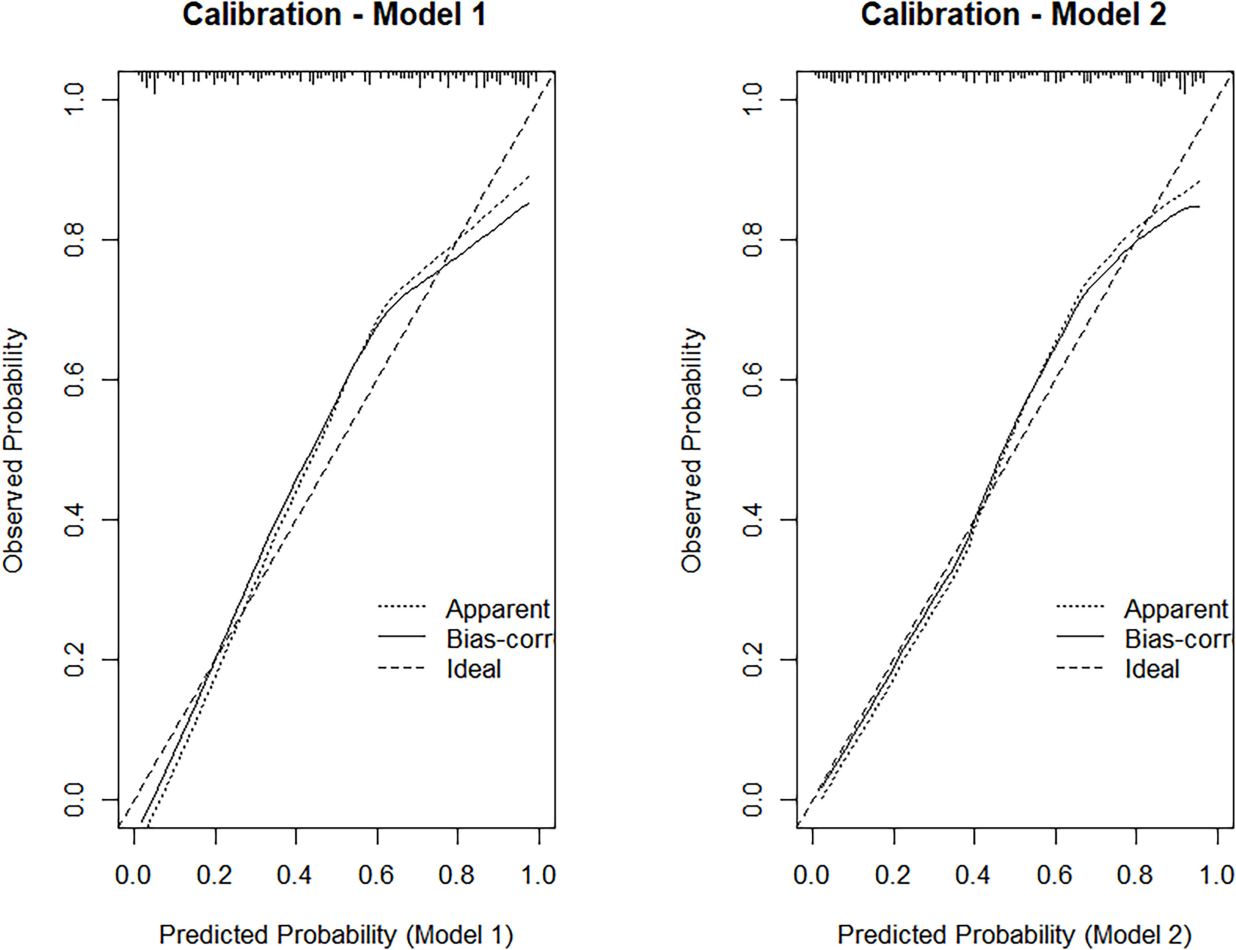
Calibration plots for Model 1 (left) and Model 2 (right), showing the agreement between predicted and observed probabilities after bootstrap correction.

Model 1 demonstrated excellent discriminative ability for predicting 90-day functional outcomes, yielding an AUC of 0.863 (Figure 1). Calibration analysis further showed strong agreement between predicted and observed probabilities, with the bootstrap-corrected calibration curve closely following the ideal reference line (Figure 2, left panel).

For Model 2, the incorporation of early neurological improvement significantly strengthened its predictive capability. ΔNIHSS was independently associated with favorable outcomes, with each five-point improvement corresponding to higher odds of good functional recovery (aOR 1.48; 95% CI, 1.11–1.97; *P* = 0.009). Other covariate associations remained consistent with those observed in Model 1 (Table 2).

**TABLE 2:** Multivariable logistic regression for 90-day good functional outcome (mRS 0–2).

Variable	Model 1 aOR (95% CI)	*P*-value	Model 2 (ΔNIHSS added) aOR (95% CI)	*P*-value
Age (per 10 years)	0.61 (0.47–0.78)	<0.001	0.63 (0.48–0.81)	<0.001
Baseline NIHSS (per five points)	0.41 (0.31–0.55)	<0.001	0.45 (0.33–0.60)	<0.001
ASPECTS (per point)	1.33 (1.10–1.64)	0.005	1.29 (1.06–1.58)	0.01
Hypertension	0.52 (0.29–0.91)	0.02	0.55 (0.31–0.97)	0.04
Diabetes	0.74 (0.38–1.40)	0.35	0.79 (0.40–1.53)	0.48
Atrial fibrillation	0.68 (0.34–1.33)	0.26	0.71 (0.35–1.41)	0.31
rtPA treatment	1.38 (0.78–2.44)	0.27	1.41 (0.77–2.56)	0.26
Mechanical thrombectomy	1.45 (0.69–2.93)	0.31	1.49 (0.71–3.01)	0.29
ΔNIHSS (per five points)	–	–	1.48 (1.11–1.97)	0.009

Model 2 achieved a discriminative performance similar to Model 1, with an AUC of 0.855 (Figure 1). Notably, calibration improved with the addition of ΔNIHSS, as reflected by a lower bootstrap-corrected calibration error (0.027 vs. 0.051), indicating more accurate risk estimation across the predicted probability range (Figure 2, right panel).

**FIGURE 3:**
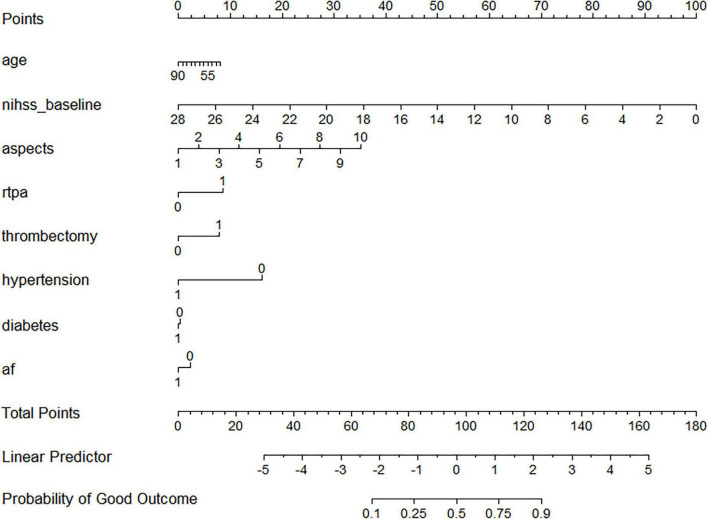
Nomogram for predicting 90-day good functional outcome (mRS 0-2) based on baseline clinical variables (Model 1).

**FIGURE 4:**
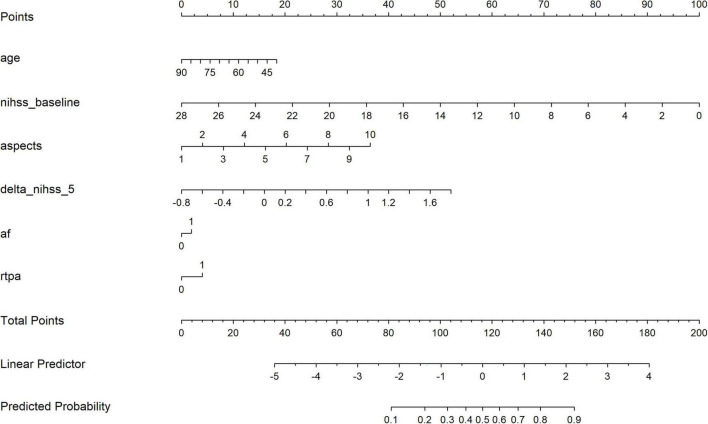
Dynamic nomogram incorporating early neurological improvement (ΔNIHSS) for individualized prediction of 90-day functional outcome.

Nomograms were constructed for both models to facilitate bedside application and individualized risk prediction (Figures 3, 4). The dynamic nomogram integrating ΔNIHSS produced smoother and more clinically interpretable risk gradients, reflecting the added prognostic value of early neurological change.

## Discussion

In this prospective cohort of 200 patients with acute ischemic stroke, early neurological improvement within 24 h emerged as a robust dynamic predictor for 90-day functional recovery (15–19). While baseline age, initial NIHSS, and ASPECTS remained powerful predictors (20, 21) of long-term outcomes—consistent with established literature—the addition of ΔNIHSS improved model calibration and enhanced clinical interpretability (15, 22). These findings support a dynamic paradigm in prognostication, emphasizing early short-term clinical response as a meaningful indicator of long-term recovery trajectory.

Our study extends current evidence in several important ways. First, prior investigations of early neurological improvement have been conducted predominantly in highly selective populations undergoing endovascular therapy, where ENI is strongly driven by angiographic reperfusion (16–18). In contrast, our cohort reflects routine clinical practice, incorporating both reperfusion-treated and medically managed patients (19, 23). This heterogeneity allowed us to demonstrate that early neurological change remained prognostically valuable even when treatment modalities varied and when ENI likely reflected a combination of biological processes, including partial reperfusion, collateral recruitment, edema resolution, and early neuroplasticity (24, 25).

Second, our comparison of baseline-only versus dynamic prediction models offers novel insight into how clinicians can integrate early change into risk stratification. Although Model 2 did not numerically increase the AUC—likely attributable to the prognostic ceiling effect of strong baseline predictors like age and admission NIHSS—the substantial improvement in calibration highlights the model’s value. In clinical practice, superior calibration ensures that predicted probabilities closely match observed outcomes, which is more critical for patient counseling than binary discrimination alone—an aspect particularly relevant when models are used for individualized prognostication. This aligns with recent methodological work emphasizing that discrimination alone is insufficient to evaluate the clinical utility of predictive models in stroke populations (21, 22, 26).

Third, our findings highlight the potential utility of ENI as a surrogate endpoint in future clinical trials (15, 27, 28). A contemporary meta-analysis reported that ENI demonstrated strong correlation with 90-day outcomes but called for more prospective validation in pragmatic settings. Our data contribute real-world evidence supporting its validity. In contexts where long-term follow-up is challenging or where early signals of treatment response are needed—such as dose-finding studies, workflow optimization, or comparative effectiveness evaluations—ΔNIHSS may serve as a useful early clinical endpoint.

Our study also underscores limitations of relying solely on baseline predictors. Age, baseline NIHSS, and ASPECTS remain foundational markers, yet they predominantly reflect initial injury burden rather than ongoing penumbral dynamics or responsiveness to therapy. The integration of 24-h neurological change offers a more complete physiological picture and may improve clinical decision-making when considering escalation of care, transition to rehabilitation, or counseling of patients and families.

The study has several limitations. First, as this was a single-center prospective cohort study, the external generalizability of our findings may be limited. In addition, the inclusion of patients receiving mixed treatment strategies, including intravenous thrombolysis and mechanical thrombectomy, may introduce clinical heterogeneity that could influence outcome estimates. Multicenter external validation will be required before the proposed dynamic prediction models can be broadly implemented in clinical practice. Furthermore, the sample size, although sufficient for logistic regression modeling, restricts our ability to explore interactions or treatment-specific ENI effects (29, 30). Imaging-based reperfusion markers (24, 31), such as mTICI or collateral grade, were not included as primary predictors due to incomplete availability in non-thrombectomy patients; future work combining physiological and imaging-based early response metrics may further refine prediction. Finally, although ΔNIHSS proved valuable, early neurological deterioration (END)—present in a minority of patients—may represent a distinct biological phenomenon, and its prognostic role warrants further investigation (25).

Despite these limitations, our findings support the increasing recognition of early neurological change as a meaningful, rapidly available, and clinically actionable dynamic predictor in AIS care. The ability to translate short-term clinical observations into long-term prognostic insight represents a significant step toward precision stroke medicine. Future multicenter validation studies and dynamic, real-time prediction tools may further enhance the integration of ENI into routine stroke workflows (19, 22, 30).

## Conclusion

In this prospective cohort of patients with acute ischemic stroke, early neurological improvement within the first 24 h was strongly associated with 90-day functional recovery. While baseline age, initial NIHSS, and ASPECTS remained important predictors, incorporating early neurological change improved overall model performance and provided more accurate individualized risk estimation. These findings validate early neurological improvement as a robust dynamic predictor rather than a mere surrogate. Integrating this short-term change into bedside models refines risk stratification by correcting probability estimates, supporting a shift from static baseline assessment to precision dynamic prognosis in routine stroke care.

## Data Availability

The raw data supporting the conclusions of this article will be made available by the authors, without undue reservation.

## Supplementary material

Supplementary Figure 1 Flow diagram of patient inclusion and exclusion.
