# Supplementary figures and images for: Early neurological improvement as a dynamic predictor for 90-day functional outcome in acute ischemic stroke: a prospective cohort study
Source: Front Med (Lausanne). 2026 Mar 9;13:1757614. doi: 10.3389/fmed.2026.1757614 (PMC13006309; doi:10.3389/fmed.2026.1757614)

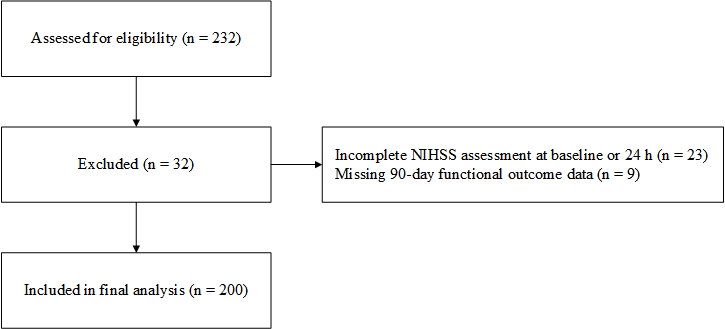

Supplement: Supplementary Figure 1 — Flow diagram of patient inclusion and exclusion. [file Image_1.jpeg]
